# The Molecular Basis of Viral Inhibition of IRF- and STAT-Dependent Immune Responses

**DOI:** 10.3389/fimmu.2018.03086

**Published:** 2019-01-08

**Authors:** Hao-Sen Chiang, Helene Minyi Liu

**Affiliations:** ^1^Department of Life Science, National Taiwan University, Taipei, Taiwan; ^2^Genome and Systems Biology Degree Program, National Taiwan University, Taipei, Taiwan; ^3^Graduate Institute of Biochemistry and Molecular Biology, College of Medicine, National Taiwan University, Taipei, Taiwan

**Keywords:** interferon, interferon-regulatory factor, signal transducer and activator of transcription signaling, interferon-stimulated gene, antiviral response, viral attenuation, viral antagonism

## Abstract

The antiviral innate immunity is the first line of host defense against virus infections. In mammalian cells, viral infections initiate the expression of interferons (IFNs) in the host that in turn activate an antiviral defense program to restrict viral replications by induction of IFN stimulated genes (ISGs), which are largely regulated by the IFN-regulatory factor (IRF) family and signal transducer and activator of transcription (STAT) family transcription factors. The mechanisms of action of IRFs and STATs involve several post-translational modifications, complex formation, and nuclear translocation of these transcription factors. However, many viruses, including human immunodeficiency virus (HIV), Zika virus (ZIKV), and herpes simplex virus (HSV), have evolved strategies to evade host defense, including alteration in IRF and STAT post-translational modifications, disturbing the formation and nuclear translocation of the transcription complexes as well as proteolysis/degradation of IRFs and STATs. In this review, we discuss and summarize the molecular mechanisms by which how viral components may target IRFs and STATs to antagonize the establishment of antiviral host defense. The underlying host-viral interactions determine the outcome of viral infection. Gaining mechanistic insight into these processes will be crucial in understanding how viral replication can be more effectively controlled and in developing approaches to improve virus infection outcomes.

## Introduction

Interferons (IFNs) were originally discovered in 1957 as proteins that interfere with virus replication (1, 2). Since then, IFNs are now divided into three sub-families: type I, II, and III with broad functions not limited to host defense against microbial infection (3–5). These secreted IFNs initiate signaling by binding distinct cell surface receptors to mount proper immune responses. Type I IFNs comprise the largest IFN family including IFN-α, IFN-β, and other subtypes. All type I IFNs bind a ubiquitously expressed heterodimeric transmembrane receptor, which is known as the IFN-α receptor (IFNAR) complex to mediate antiviral effects of type I IFNs (3). IFN-γ is the sole type II IFN largely secreted by innate lymphocytes and T cells that binds to IFN-γ receptor (IFNGR) complex and activates several immune responses to intracellular pathogens (5). Distinct from type I and type II IFNs, type III IFNs are recently discovered and consist of IFN-λ1 (interleukin-29 [IL-29]), IFN-λ2 (IL-29A), IFN-λ3 (IL-28B), and IFN-λ4 (6). They engage the mucosal surface-abundant receptor complex, IFN-λ receptor (IFNLR, also known as IL-28R) that consists of two subunits: IFNLR1 and IL10R2 in the initiation of protection against viral infection at mucosal barriers (4).

Upon virus infection, IFNs are immediately induced by the recognition of pathogen-associated molecular patterns (PAMPs) through cytoplasmic and endosomal pattern-recognition receptors (PRRs) or by cytokine-receptor binding (7). The IFN-regulatory factor (IRF) family proteins are transcription factors with critical and diverse roles that connect the sensing of microbial signatures to the expression of IFNs and pro-inflammatory cytokines as well as innate immune responses (8–10). After IFN binding and receptor dimerization, all IFNs induce IFN-stimulated gene (ISG) expression for effective antiviral responses through the activation of IFN receptor-associated Janus kinase-signal transducer and activator of transcription (JAK-STAT) pathway (11). As obligate intracellular microbes, viruses must engage with the host throughout their replication; it is therefore unsurprised that pathogenic viruses often antagonize IFN responses to establish successful infections by targeting the aforementioned pathways.

In this review, we critically explore the current understanding of IRF and STAT family proteins in host antiviral immune responses activated by IFNs; we also examine how pathogenic viruses have evolved various mechanisms to suppress IRF- and STAT-mediated signaling.

## IRFS in the Production of IFNS During Virus Infection

**Figure 1:**
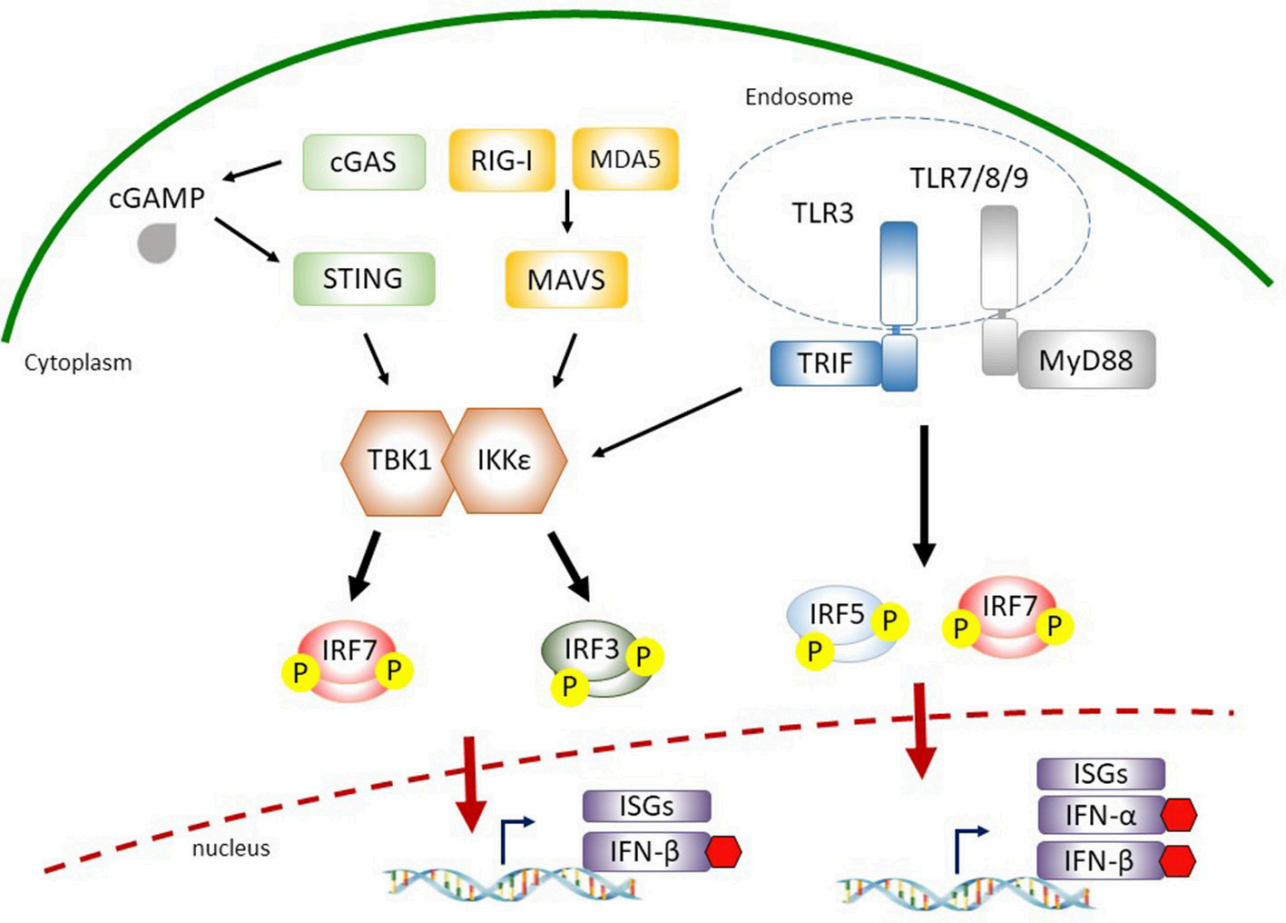
Interferon (IFN)-regulatory factors (IRFs) involved in cytosolic nucleic acid sensing and endosomal Toll-like receptor (TLR) signaling. During virus infection, retinoic acid-inducible gene I (RIG-I) or melanoma differentiation-associated gene 5 (MDA5) recognize cytosolic double-stranded RNA and recruit the adaptor protein mitochondria antiviral signaling protein (MAVS), which leads to the activation of TANK-binding kinase 1 (TBK1)/IκB kinase-ε (IKKε). Cytosolic double-stranded DNA is detected by cyclic-GMP-AMP (cGAMP) synthase (cGAS) or other receptors (such as DEAD-box helicase 41 (DDX41), gamma-IFN-inducible protein 16 (IFI16), not shown) to induce stimulator of IFN genes (STING)-mediated TBK1 and IKKε activation. Activated TBK1/IKKε then phosphorylate IRF3 and IRF7 that translocate into the nucleus for the induction of IFN-β. The sensing of viral pathogen-associated molecular patterns (PAMPs) by endosomal TLR3 or TLR7/8/9 leads to the phosphorylation and activation of IRF5 and IRF7 through adaptor proteins TIR-domain-containing adapter-inducing IFN (TRIF) or myeloid differentiation primary response 88 (MyD88), respectively, for the expression of type I IFNs.

The mammalian IRF family proteins are structurally related transcription factors consisting of nine members: IRF1, IRF2, IRF3, IRF4 (also called ICSAT [IFN consensus sequence-binding protein for activated T-cells], LSIRF [lymphocyte-specific IRF], PIP [PU.1-interacting protein]), IRF5, IRF6, IRF7, IRF8 (also referred to ICSBP [IFN consensus sequence-binding protein]), and IRF9 (also known as ISGF3γ [IFN-stimulated gene factor 3γ]) (9). Among nine IRFs, IRF1, IRF3, IRF5, and IRF7 play a pivotal role in the induction of IFN gene transcription during viral infection (12, 13). IRF2 and IRF4 have been implicated in the suppression of type I IFN signaling (14–16). All IRF family proteins possess two conserved functional domains: an amino (N)-terminal DNA-binding domain (DBD) and a carboxy (C)-terminal IRF-associated domain (IAD) (17). DBD is characterized by five conserved tryptophan residues that forms a helix-turn-helix structure and recognizes consensus DNA sequence known as IFN-stimulated response element (ISRE) (18). In contrast to N-terminal regions, the C-terminal regions of IRFs display a broad diversity. Two types of IAD have been identified: IAD1 and IAD2 (19). While IAD1 is conserved in all IRFs except IRF1 and IRF2, IAD2 is shared only by IRF1 and IRF2 (20). The C-terminal regions of IRFs are also involved in the interactions with other IRF family proteins or transcription factors/co-activators that are critical for the induction of IFN (21, 22). For example, IRF3 forms a complex with CREB binding protein (CBP)/p300 histone acetyltransferase (HAT) through the IAD1 domain for the induction of *Ifnb1* transcription in response to virus infection (21). In the following sections, we discuss the distinct contribution of IRFs to type I IFN induction through cytoplasmic and endosomal PRR signaling cascades (Figure 1).

### IRF3 and IRF7 Are the Master Regulators of Type I IFN Expression in RLR Signaling

During virus infection, type I IFNs are produced in infected cells via the recognition of viral PAMPs by binding to specific PRRs, such as cytosolic retinoic acid-inducible gene I (RIG-I)-like receptors (RLRs) and transmembrane Toll-like receptors (TLRs) resulting in the activation of downstream IRF3 and IRF7 pathways (7, 23). Several RNA viruses directly enter the cytoplasm where they are detected by RLR family members: RIG-I and melanoma differentiation-associated gene 5 (MDA5) (24). Ligand recognition results in the recruitment of RIG-I and MDA5 to the mitochondria where they interact with mitochondria antiviral signaling protein (MAVS) through the N-terminal caspase recruitment domain (CARD) domains in RLRs and MAVS. This association relays signals to the downstream TANK-binding kinase 1 (TBK1) and IκB kinase-ε (IKKε) that phosphorylate IRF3 and IRF7 (24).

IRF3 is a constitutively expressed but tightly regulated transcription factor in the cytoplasm. It presents in an inactive form due to its auto-inhibitory mechanisms (25). Virus infections induce specific IRF3 phosphorylation that leads to its dimerization with itself or with IRF7 and forms a complex containing CBP/p300 and other coactivators followed by translocation into the nucleus for the expression of IFN-β (26). The activation process of IRF7 is similar to that of IRF3 in response to viral PAMPs. However, in contrast to constitutively expressed IRF3, the basal expression level of IRF7 is minimum but is strongly induced by type I IFN-mediated responses in an autocrine feedback loop after virus infection (discussed in detail below) (9). Moreover, a recent study from IRF3/IRF5/IRF7 triple knockout mice suggests that in addition to IRF3 and IRF7, IRF5 is also a key transcriptional factor responsible for RLR- and MAVS-mediated type I IFN expression (27).

### Contributions of IRFs to the Induction of Cytosolic DNA-Mediated and TLR3/7/8/9-Mediated Type I IFN

Similar to the involvement of RLR-mediated type I IFN expression, IRF3 and IRF7 also contribute to the signaling pathways downstream of cytosolic DNA sensing and endosomal DNA/RNA recognition for the inductions of IFN-α and IFN-β during virus infection (7). Among several known cytosolic DNA sensors for the detection of viral infection, cyclic-GMP-AMP (cGAMP) synthase (cGAS) is the most recently identified (28). Upon viral DNA binding, cGAS catalyzes the production of cGAMP from ATP and GTP, a second messenger that binds and activates the endoplasmic reticulum membrane protein stimulator of IFN genes (STING) for the production of type I IFN (28, 29). STING functions as an adaptor protein that promotes TBK1-dependent IRF3/7 phosphorylation (30–33).

Transmembrane TLR3, TLR7/8, and TLR9 are the most well characterized PRRs for the recognition of viral PAMPs located in the endosomal compartments (34). TLRs initiate shared and distinct signaling pathways by recruiting different adaptor molecules for type I IFN expression. TLR3 recognizes viral dsRNA and utilizes TIR-domain-containing adapter-inducing IFN (TRIF) as an adaptor to recruit downstream TBK1, resulting in IRF3/7 phosphorylation and type I IFN production. Upon the engagements with viral ssRNA and unmethylated CpG DNA motifs by TLR7/8 and TLR9, respectively, these TLRs signal through myeloid differentiation primary response 88 (MyD88) to activate IKKα- or IKKβ-dependent phosphorylation and activation of IRF7 or IRF5, allowing the production of type I IFNs (35–37). Taken together, these studies highlight the importance of IRF3/5/7 phosphorylation and activation in the downstream of cytoplasmic/endosomal PRR signaling leading to type I IFN expression during virus infection.

## IRFS and Stats in IFN-Mediated Innate Immune Responses

**Figure 2:**
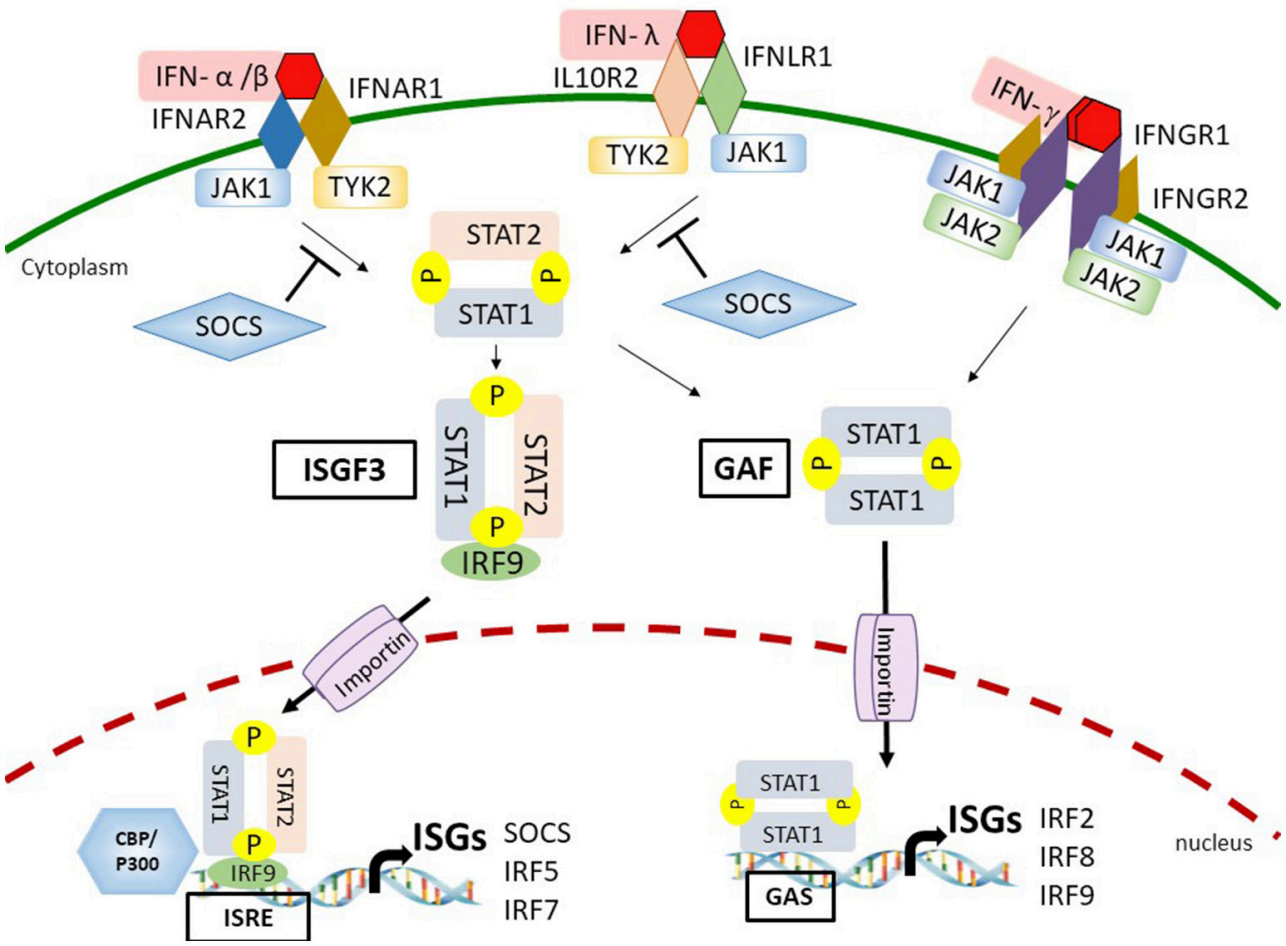
Interferon (IFN)-dependent IFN stimulated gene (ISG) transcription through the Janus kinase-signal transducer and activator of transcription (JAK-STAT) signaling. By binding to the IFN-α receptor (IFNAR) or IFN-λ receptor (IFNLR), type I/III IFNs activate the JAK-STAT pathway leading to the formation of IFN-stimulated gene factor 3 (ISGF3) and gamma-IFN activation factor (GAF) complexes. The ligation between IFN-γ and IFN-γ receptor (IFNGR) also activate the GAF complex. ISGF3 and GAF complexes then translocate into the nucleus mediated by importins and recruit additional coactivators, such as CREB binding protein (CBP)/p300 on the IFN-stimulated response element (ISRE)- or gamma IFN activated sequence (GAS)-containing promoters to stimulate expression of a distinct group of ISGs. Eventually, a set of ISGs are produced and amplify the IFN response. IFN-induced suppressor of cytokine signaling (SOCS) proteins inhibit JAK-STAT signaling by binding to phosphorylated tyrosine residues on either JAK1 or tyrosine kinase 2 (TYK2).

Mammalian immune systems utilize IRFs, STATs and IFNs to integrate and process distinct signals to orchestrate host antiviral immunity. This has been proven in several studies utilizing genetically-modified mice that lack IFNAR, IFNGR, IFNLR, STAT1, or STAT2, respectively. These gene-knockout mice are highly susceptible to virus infections due to the impaired IFN signaling and responses (38–42). In the following sections, we examine the current understandings of how IFNs initiate antiviral immune responses via binding to their cognate heterodimeric receptors through downstream canonical JAK-STAT signaling (Figure 2).

### Canonical IFN-Activated JAK-STAT Pathway

Almost all cell types respond to type I and type II IFNs for effective antiviral immunity (43, 44). However, the specific type III IFN receptor subunit IFNLR1 is mainly expressed on epithelial cells and immune cells, such as neutrophils that provides the first line of antiviral defense at the mucosal surfaces of gastrointestinal and respiratory tracts (42, 45). The ligation between IFN and IFNR results in receptor dimerization or oligomerization that allows the transphosphorylation of receptor-associated JAK on tyrosine residues. Subsequently, activated JAKs induce tyrosine phosphorylation of IFNR cytoplasmic tails where provides the binding sites for C-terminal Src-homology-2 (SH2) domains of STAT proteins. The STATs are then recruited to the JAKs followed by phosphorylation at a tyrosine residue (46). In the canonical pathway of type I IFN-mediated and type III IFN-mediated signaling, the phosphorylation of STAT1 on tyrosine 701 and STAT2 on tyrosine 690 leads to the formation of STAT1/STAT2 heterodimer that interacts with IRF9 to form the IFN-stimulated gene factor 3 (ISGF3) complex. ISGF3 complex then redirects and translocates into the nucleus to trigger ISG expressions by binding to ISRE and recruiting additional coactivators, such as CBP/p300 on the promoters of a distinct group of target genes (44, 47–49). In addition to forming the ISGF3 complex, all types of IFNs are able to induce a STAT1/STAT1 homodimer, known as gamma IFN–activation factor (GAF) that activates ISG transcription by direct binding to the gamma IFN activated sequence (GAS)-containing genes (4, 44). Besides the STAT1/STAT2 heterodimer and STAT1/STAT1 homodimer, type I IFNs can also activate STAT3/STAT3, STAT4/STAT4, STAT5/STAT5, and STAT6/STAT6 homodimers as well as STAT1/STAT3, STAT1/STAT4, STAT1/STAT5, STAT2/STAT3, and STAT5/STAT6 heterodimers. All of these homodimers and heterodimers can bind to GAS and drive the expression of GAS-containing ISGs (44).

### ISGs and Regulation of JAK-STAT Family Proteins

ISGs are proteins present at baseline but are enhanced upon virus infection in JAK-STAT dependent pathways. A subset of ISGs are well-characterized for their direct antiviral activities. For example, IFN-induced proteins with tetratricopeptide repeat 1–3 (IFIT1–3), viperin, and myxovirus resistance 1 (Mx1) can all inhibit virus replication (50). JAK2, STAT1, STAT2, and IRF9 belong to another subset of ISGs that amplify JAK-STAT signaling to reinforce IFN responses (3). Moreover, ISGs, such as RIG-I, cGAS, IRF5, and IRF7 can further prime cells for increased detection of viral PAMPs and upregulated IFN expressions (51). Interestingly, the expression of subsets of ISGs can also be induced directly by IRFs in a pathway that is independent of JAK-STAT signaling (52). For example, IRF3 initiates the expressions of IFN-stimulated gene 15 (ISG15) and sterile alpha motif and HD domain containing protein 1 (SAMHD1) as the first responders in antiviral immunity (53, 54). Similar to IRF3, IRF1 mediates a rapid IFN-independent antiviral response downstream of RLR adaptor protein MAVS localized specifically on peroxisome (55–57). IRF7 can regulate ISG expression in the absence of IFN signaling as well (50, 58).

In addition to JAK2. STAT1, STAT2, IRF9 themselves, several other ISGs are also implicated in the regulation of JAK-STAT signaling. Suppressor of cytokine signaling (SOCS) proteins including SOCS1 and SOCS3 inhibit JAK-STAT signaling by binding to phosphorylated tyrosine residues on either JAK1 or tyrosine kinase 2 (TYK2) (59). Another ISG: the ubiquitin-specific peptidase 18 (USP18) suppresses JAK-STAT signaling induced by type I IFN at the level of IFN receptor. USP18 specifically interacts with the IFNAR2 subunit to inhibit the interaction between JAK and the IFN receptor (60). Furthermore, recent findings indicate that type I IFN-induced STAT3 cooperates with phospholipid scramblase 2 (PLSCR2) to interact with STAT2 and suppress type I IFN response (61, 62). It would be critical to further determine whether STAT family members also interact with other proteins for the regulation of JAK-STAT pathways.

### Protein Regulators of STAT Family Proteins

As STAT family proteins are essential signaling mediators, their activation are tightly-regulated by several mechanisms in order to downregulate IFN-mediated antiviral response (63). STATs reside in an inactive form in the cytoplasm but are activated and translocate into the nucleus in response to IFN signaling. Several nucleocytoplasmic transport factors are essential for the nuclear import of phosphorylated STATs. For example, importin-α5 (also called karyopherin α1[KPNA1]) regulates nuclear import of STAT1 (64, 65). Moreover, STAT3 nuclear import is mediated by importin-α3 (66). Interestingly, the presence of phosphorylated STATs in the nucleus is transient. STAT1 can be dephosphorylated in the nucleus and actively export to the cytoplasm by the chromosome region maintenance 1 (CRM1) export receptor in a nuclear export signal (NES)-dependent manner (67). Recently, a metheyltransferase SET domain-containing protein 2 (SETD2) has been identified as a critical type I IFN signaling amplifier. Although the expression of SETD2 itself is not upregulated by type I IFN, SETD2 enhances the methylation of STAT1 on K525 as well as ISG expressions for antiviral immunity (68). However, the detail mechanism underlying the regulation and selectivity of SETD2-mediated ISG expression has not been fully explored.

## Viral Regulation and Evasion of the IRF- and STAT-Dependent Anti-Viral Pathways

In order to establish successful infections, viruses have evolved a variety of strategies to counteract host antiviral innate immunity. The infection outcome is determined by the race between the kinetics of virus replications and the competence of antiviral gene expression levels at the early-onset of the infection. Studies of virus-host interactions have revealed infection-induced signaling pathways that result in IRF and STAT activations as the major targets of regulation and evasion of the host anti-viral responses. The molecular mechanisms by which viruses target IRFs and STATs are highly diverse, including inhibition of IRF/STAT expressions, disruption of the post-translational modifications, alterations in the localizations, prevention of transcriptional complex formation, and promoting the degradation of IRFs and STATs.

### Disruption of the IRF/STAT Post-translational Modifications

For rapid response to the viral infections, the activation of antiviral innate immunity in mammalian cells is largely controlled by the post-translational modifications of the PRR, the downstream signaling adaptor proteins, as well as the key transcription factors, IRFs and STATs. Phosphorylation of IRF3, IRF7, STAT1, and STAT2 are highly critical for their downstream transcriptional activation, and therefore these phosphorylation events are commonly targeted by viruses. By directly promoting the dephosphorylation of IRFs and STATs or indirectly inhibiting upstream kinase activities, the activation of these transcription factors are then controlled by the invading viruses.

Vaccinia virus (VACV) encodes a late gene VH1, which is packaged into the virion and is a dual-specificity phosphatase (69). VH1 was later found to have immediate effects against host antiviral activities by directly dephosphorylating STAT1 at Tyr701 and Ser727 to reduce STAT1 activation (70, 71). Besides, VACV encodes another virulence factor, C6, which binds to the IRF3/7 kinase TBK-1 and interferes the phosphorylation and activation of IRF3 and IRF7 (72). Several other DNA viruses also have similar mechanisms to target TBK1 and/or IKKε to inhibit the phosphorylation of IRF3. It has been recently reported that the VP24 of herpes simplex virus (HSV) can target TBK1/IKKε to inhibit the phosphorylation of IRF3 (73, 74). Also, several viruses have evolved viral products that may interfere the interaction between STAT1 and JAK/TyK, e.g., the NS5A protein of Hepatitis C virus can physically interact with STAT1 to interfere the phosphorylation of STAT1 at Tyr701 (75, 76). Another example is the NSP1 protein encoded by Rotavirus, of which the expression alone may block STAT1 phosphorylation and activation (77). Ebola virus VP35 prevents the TBK1-dependent phosphorylation of IRF3 (78, 79). Marburg virus, which is closely related to Ebola virus, encodes a matrix protein VP40 to antagonize the phosphorylation of both STAT1 and STAT2 (80).

In addition, viruses may regulate IRF/STAT phosphorylation indirectly by promoting the expression of the negative regulators of IRF/STAT kinases, such as suppressor of cytokine signaling (SOCS) family, to minimize the induction of ISGs. It has been reported that Hepatitis B virus (HBV) infection as well as Hepatitis C virus (HCV) core protein may induce the expression of suppressor of cytokine signaling 3 (SOCS3) (81, 82). The e antigen of HBV, HBeAg, also stimulates the expression of suppressor of cytokine signaling 2 (SOCS2) (83). The induction of SOCS family subsequently impairs IFN/JAK/STAT signaling and therefore attenuating the activation of STAT1, which may contribute to the establishment of persistent infections of HBV and HCV.

### Virus-Induced Proteolysis or Degradation of IRF/STAT Proteins

Many viruses, such as picornaviruses and flaviviruses, encode viral proteases for viral replications. In addition to their essential roles in the virus life cycles, the viral proteases often target host proteins involved in IFN induction and response pathways to escape the host antiviral innate immunity (84). The 3C proteases (3Cpro) encoded by enterovirus (EV) 71 and EV68 disturb the expression of type I IFN and ISGs by directly cleaving IRF7 (85, 86). Porcine deltacoronavirus nsp5 cleaves STAT2 to antagonize type I IFN responses (19). These proteolytic events of IRFs and STATs often lead to the degradation of these transcription factors. For example, besides cleaving IRF7, the 3Cpro of enterovirus 71 also targets IRF9 for proteolytic degradation (87).

Conversely, a variety of viral proteins may also promote the proteasome-dependent or lysosome-dependent protein degradation of IRFs and STATs. It has been shown that the Vpu accessory protein of human immunodeficiency virus (HIV) mediates the depletion of IRF3 through lysosomal degradation or caspase-dependent cleavage (88, 89). HIV YU2 mutant which lacks the expression of Vpu could not control the activation of IRF3 upon infection (89). The NSP1 of rotavirus, a putative E3 ubiquitin ligase, mediates the degradation of cellular factors, including IRF3, IRF5, IRF7, and IRF9 but not IRF1, through recognizing the common IAD1 domain of these IRFs (90, 91).

Several members of the *Paramyxoviridae*, such as parainfluenza viruses, have developed strategies to target either STAT1 or STAT2 for degradation. The expression of a single viral protein, human parainfluenza virus type 2 (hPIV-2) V protein, may inhibit the type I IFN response by inducing the proteolytic degradation of STAT2 (92), and Newcastle disease virus (NDV) also encodes a V protein which can target STAT1 for degradation. The V protein of canine parainfluenza virus 5 (also known as *simian virus 5*) degrades both STAT1 and STAT2 in a proteasome-dependent manner (93). The NS5 proteins of flaviviruses, including dengue virus (DENV) and Zika virus (ZIKV), also share the common characteristics to target STAT2 for proteasome-dependent degradation (94–97).

### Re-localization of IRF/STATs by Viral Proteins

After phosphorylated, the nuclear translocation of the activated IRFs and STATs is another key step to induce the transcription of downstream genes, and this process is largely dependent on the cellular nuclear import and export machineries, including importin and CRM-1 proteins. Hence, viruses have developed various strategies to negatively regulate IFN induction and response pathways by altering the localizations of activated IRFs and STATs. Ebola virus VP24 binds to the α5 and α6 subunits of importin, which are the essential components of the nuclear transporter, to block the nuclear translocation of phosphorylated STAT1 (98, 99). EV71 suppresses IFN responses by blocking STAT1 signaling through inducing importin-α5 degradation in a caspase-3-dependent manner (100). As described previously, nuclear STAT1, STAT2, and IRF9 cycle back to the cytoplasm in a CRM1-dependent nuclear export manner. Certain viral proteins, such as the nsp2 of chikungunya virus, can promote the nuclear export of STATs (101, 102). During Nipah virus infection, the V protein can directly bind to STAT1 and STAT2 in the cytoplasm, and the N protein of Nipah virus restricts the complex formation of STAT1/STAT2, which along with the CRM1-dependent nuclear export of STAT1 and STAT2 additively result in the accumulation of STAT1 and STAT2 in the cytoplasm (103–105).

Viruses may also encode proteins which can directly bind to IRFs and STATs and maintain their cytoplasmic distribution. The C proteins of hPIV-1, which belongs to the *Paramyxoviridae*, blocks IFN signaling by interacting and retaining STAT1 and STAT2 in the perinuclear region in the infected cells (106). Similarly, the C protein of Sendai virus, also known as murine paramyxovirus, binds p-STAT1 to inhibit STAT1 dimerization and nuclear translocation (107, 108). Measles virus, which also belongs to the *Paramyxoviridae*, does not inhibit the tyrosine phosphorylation of STAT1 and STAT2 but encodes a viral protein, V protein, which directly interacts with STAT1, STAT2, and IRF9 in the cytoplasm to prevent their nuclear translocation (109). In addition to the *Paramyxoviridae*, the monkey rotavirus and human rotavirus Wa strain also do not restrict the activation phosphorylation of STAT1 and STAT2 but retain these transcription factors in the cytoplasm (110). HSV-1 encodes several IFN antagonists, including ICP0, which inhibits IRF3 nuclear accumulation but not IRF3 phosphorylation (111). Human papilloma virus (HPV) E7 protein interacts with IRF9 in the cytoplasm and subsequently inhibits the nuclear translocation of IRF9 as well as the formation of ISGF3 (112).

### Interference of the Transcriptional Complex Formation of IRFs and STATs

After translocation into the nucleus, the activated IRFs and STATs will then bind to the promoter region on the chromosomal DNA and recruit other transcription activators to initiate the transcription of downstream genes. A distinct group of viral proteins, viral homologs to cellular IRFs, known as vIRFs, are reported to be encoded by Kaposi's sarcoma-associated herpesvirus (KSHV) and the rhesus macaque rhadinovirus (RRV). KHSV vIRF3 interacts with host IRF5 and IRF7, and vIRF3 can suppress IRF7 DNA binding activity to inhibit IFN-α production (113, 114). Furthermore, KHSV vIRF1 binds to p300 and interferes with CBP/p300-IRF3 complex formation as well as the HAT activity of p300, and thus prevents IRF3-mediated transcriptional activation (115). HSV-1 also utilizes a similar strategy to abrogate CBP recruitment by IRF3 through the viral protein VP16 (116). RRV-encoded R6 is a virion-associated vIRF, which is capable to prevent IRF3/CBP complex docking to the IFNβ promoter region to minimize the induction of type I IFN expression upon RRV infection (117).

Besides vIRFs, other viral proteins have also been reported to regulate the DNA-binding activities and transcriptional complex formation. For example, Porcine bocavirus (PBoV) NP1 protein inhibits the DNA-binding activity of IRF3 and the DNA-binding activity ISGF3 through interactions with the DNA-binding domain of IRF9 (118, 119). The nsp1 of porcine epidemic diarrhea virus (PEDV) and the nsp1 α subunit of porcine reproductive and respiratory syndrome virus (PRRSV) both suppress the type I IFN production by promoting the proteasome dependent degradation of CBP (120, 121).

### Inhibition of IRF and STAT Protein Expression

As described in ISGs and Regulation of JAK/STAT Family Proteins, the protein expression levels of various of the IRFs and STATs are upregulated in response to viral infections and IFN signaling, such as IRF7 and STAT1 (51), to form a positive feedback loop for the enhancement of antiviral activities. Therefore, disturbing the activation of basally expressed endogenous IRFs and STATs not only directly impairs the induction of the initial round of antiviral gene expressions but also prohibits the magnification of the antiviral responses against virus replication. Without sufficient and efficient antiviral gene expression in the infected cells, the viruses may competently replicate and produce viral progenies to infect neighboring cells and therefore establish a successful infection.

Some viruses directly inhibit the expression of IRFs at the transcriptional level, e.g., Epstein-Barr virus (EBV) BRLF1 inhibits the transcription of IRF3 and IRF7 (122). Another major mechanism for herpes viruses to curtail the expression of IRFs and STATs is virion host shut-off (VHS), which is mediated by the tegument protein UL41 (123). It has been shown that through its own endoribonuclease activity, HSV VHS selectively promotes the degradation of host mRNAs made before infection, including the mRNA of ISGs (124, 125). Many of the RNA viruses, including *Caliciviridae, Coronaviridae, Picornaviridae*, O*orthomyxoviridae, Reoviridae*, and many others, have strategies to induce host translational shut-off and thus prevent the infected cells to synthesize new peptides and proteins, including those IFN-stimulated IRFs and STATs. Viruses may also upregulate certain miRNA to tune the expression of factors involved in the activation of IRFs and STATs. A recent report showed that miR-373, which reduces the expression of both JAK1 and IRF9, is upregulated by HCV infection to suppress the response to IFNs in the infected cells (126).

## Conclusion and Perspectives

In the past decades, the inductions and responses of IFNs have been much revealed. The regulatory network of antiviral innate immunity including IFNs and many other cytokines is extremely complex in the mammalian cells. Since viruses carry much less genetic information than the eukaryotic cells, analyses of which cellular factors are targeted by viral products to dampen the innate immunity pathway provide us with great means to identify the crucial signaling molecules for mammalian antiviral activities. The critical role STAT1 in antiviral immunity is well-pronounced since viruses have developed numerous strategies to target STAT1 as a result of evolution. Notably, the development of STAT1 KO mice as *in vivo* animal models for viral infections has provided valuable tools for future virology and immunology studies (127–129).

As we reviewed in this article, all pathogenic viruses have multiple strategies to antagonize the host antiviral innate immunity. Intriguingly, several viruses selectively inhibit the type I IFN-induced but not type II IFN-induced STAT1 phosphorylation, such as ZIKV (130, 131). How this is beneficial to the virus life cycle remains to be further investigated. With better understanding the molecular mechanisms behind, future developments of antiviral agents and vaccines will be accelerated.

## Author Contributions

All authors contributed equally to this work. H-SC and HL conceptualized, wrote, and edited the manuscript.

### Conflict of Interest Statement

The authors declare that the research was conducted in the absence of any commercial or financial relationships that could be construed as a potential conflict of interest. The handling Editor declared a shared affiliation, though no other collaboration, with the authors H-SC and HL.
